# Linking Personality and Performance in California Sea Lions (*Zalophus californianus*) During Computerized Cognitive Enrichment

**DOI:** 10.3390/ani15203007

**Published:** 2025-10-16

**Authors:** Amber Ramos, Kelley Winship

**Affiliations:** 1 School of Health in Social Science, University of Edinburgh, Edinburgh EH8 9YL, UK; 2 Sea Life Park Hawai’I, Honolulu, HI 96795, USA; 3 National Marine Mammal Foundation, San Diego, CA 92106, USA; kelley.winship@nmmf.org

**Keywords:** California sea lion, computerized task, cognitive enrichment, personality, individual differences

## Abstract

**Simple Summary:**

Cognitive enrichment can improve the well-being of animals in professional care, but not every animal is motivated by the same kinds of challenges. In this study, California sea lions were assessed to see whether differences in personality were linked to how they engaged with a computerized game. Trainers who knew each animal well rated their personalities using a list of common traits, and the animals then interacted with a computerized device that recorded measures such as response speed and the number of button presses used to complete tasks. The analysis showed patterns suggesting that personality was related to style of play. Some trait groupings were associated with faster responses or greater efficiency, while others were linked with changes in performance across sessions. These findings suggest that enrichment can be personalized to align with the preferences and tendencies of each animal. Although based on a small sample and limited data outputs, the results indicate that tailoring cognitive activities to individual differences may support enrichment design and overall animal welfare.

**Abstract:**

Cognitive enrichment is increasingly recognized as an important component of marine mammal welfare, offering animals opportunities for choice, problem solving, and sustained engagement. Personality research has also shown that stable individual differences can influence how animals interact with enrichment, training, and their environment. This study examined how trainer-assessed personality traits related to interaction patterns with a computerized enrichment system in California sea lions (*Zalophus californianus*). Using validated personality surveys, reliability testing, and hierarchical clustering, trait structures were derived and compared with objective, system-generated gameplay measures. Analyses revealed meaningful associations that emphasize the value of considering temperament when designing enrichment and welfare programs. By integrating personality assessment with technology-based enrichment systems, this work demonstrates how individualized approaches can enhance welfare and provide new insights into enrichment design and animal cognition. Although limited in scope and sample size, the findings suggest that computerized enrichment has potential as both a welfare tool and a research platform, with value in considering personality and individual variation for broader applications across species and settings.

## 1. Introduction

Human–animal relationships, individualized care, and innovative enrichment systems are central to advancing the welfare of marine mammals. California sea lions (*Zalophus californianus*), like many marine mammals, are highly intelligent and socially complex [1,2,3]. California sea lions possess advanced problem-solving skills and social learning capacities that have made them valuable subjects in cognitive research and public education [4,5]. These same abilities have also supported their use in specialized programs such as the U.S. Navy Marine Mammal Program, where they have been trained for object recovery and harbor security tasks [6]. These traits also mean they are particularly sensitive to the quality of their environment and their daily interactions with caregivers. Care strategies must therefore address not only physical needs but also cognitive, social, and behavioral needs.

Cognitive enrichment plays a particularly important role in this context. Defined as activities or challenges designed to stimulate mental abilities and problem-solving skills, cognitive enrichment can promote psychological well-being and strengthen the human–animal relationship by creating collaborative and rewarding experiences [2,7,8,9,10,11]. Earlier enrichment programs often emphasized physical activity or food rewards, but more recent studies reinforce the value of cognitive challenge, particularly opportunities that provide animals with choice, problem-solving, and a sense of control [2]. This shift parallels the growth of individualized welfare assessments for marine mammals, which stress the importance of tailoring opportunities to each animal’s abilities and preferences [12]. For California sea lions specifically, cognitive enrichment has included tasks ranging from simple discrimination exercises to more complex computerized activities, each placing different demands on problem solving and learning [2,13,14].

**Figure 1:**
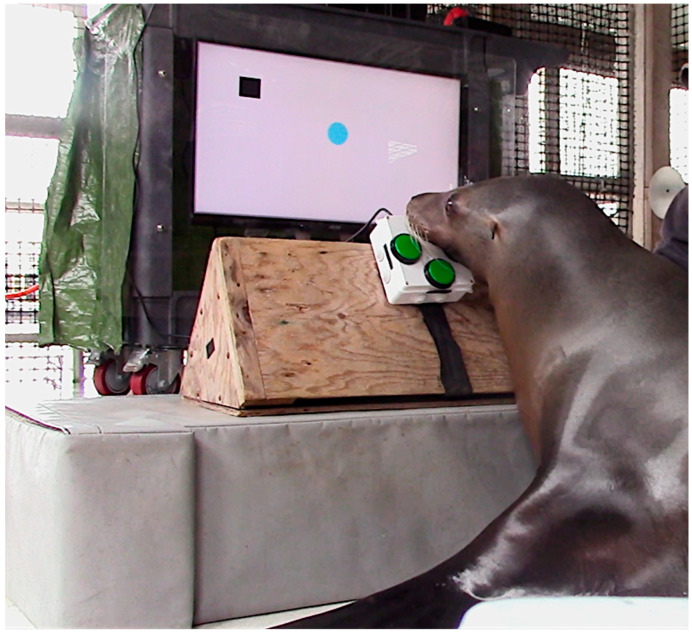
A sea lion engaging in the initial training game, navigating a blue cursor towards a black target (box), using a four-button controller.

**Figure 2:**
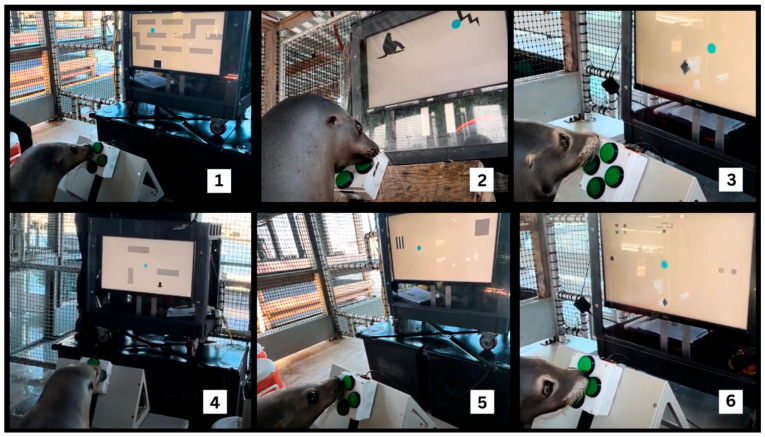
A sea lion interacting with different computer-based games, displayed from left to right, top to bottom: (1) a maze navigation game; (2) a match-to-sample task; (3) a “chase” task involving pursuit of a moving target with both patterned and random movements; (4) a similar chase task using a human-operated moving target with obstacles; (5) a game that measures visual acuity; and (6) a selection game that allows the player to choose among all previously mastered games from a menu.

One example of this approach is the Enclosure Video Enrichment (EVE) system, a computerized interface designed to allow sea lions to engage with video-based challenges outside of routine training and husbandry [14]. Developed through collaborative design and stepwise training, EVE was intended to provide meaningful cognitive engagement for Navy sea lions once they return from their tasking at sea. The EVE system requires participating animals to manipulate a four-button controller to complete a series of on-screen tasks (Figure 1). Current games include: (1) a training game in which the player learns to guide a cursor into progressively smaller targets requiring increasing precision; (2) a maze navigation game; (3) a match-to-sample task; (4) a “chase” task involving pursuit of a moving target with both patterned and random movements; (5) a similar chase task using a human-operated moving target with obstacles; (6) a game that measures visual acuity; and (7) a selection game that allows the player to choose among all previously mastered games from a menu [15] (Figure 2). However, the gameplay data analyzed in this study pertain exclusively to performance in the initial training game. The system also generates detailed records of animal interactions, turning enrichment into both a welfare tool and a research platform. Regular interaction with EVE has been associated with reduced days of clinical illness, greater consistency in voluntary participation, and improved food consumption, linking cognitive challenge to measurable improvements in health and welfare [16]. Its adaptability has also been demonstrated in other species and settings [17,18,19].

While such enrichment approaches can be beneficial, effectiveness may differ across individuals, reflecting variation in motivation, experience, and behavior. Reviews often suggest that enrichment may be more effective when designed not only for a species, but also with the preferences and capacities of individuals in mind [2,8,10]. An individualized approach is essential for ensuring that enrichment remains engaging and beneficial over time [11,20]. Examining personality provides a useful framework for interpreting and incorporating individual differences.

Personality, broadly defined as the enduring patterns of behavior and traits that shape an individual’s adjustment to life [21], has been shown to remain relatively stable across time in both humans and animals [22,23]. In marine mammals, personality traits have been documented across developmental stages and environments [24,25]. Research on pinnipeds, including sea lions (*Zalophus californianus*) and harbor seals (*Phoca vitulina*), has documented stable traits such as boldness and behavioral regularity across situations [26]. Personality traits have practical implications for welfare because they influence how animals learn, respond to training, and engage with enrichment opportunities [2,10,27]. The Five-Factor Model (openness, conscientiousness, extraversion, agreeableness, neuroticism) has been applied across species [1,22,23]. Traits within this framework relate to engagement, exploration, and problem-solving across taxa [28,29,30], and incorporating personality into enrichment or training can better align challenges with individual capacities [2,31].

Effective enrichment should not rely on species-level assumptions alone. It must also account for measurable individual differences that influence engagement and outcomes. Building on prior work with the EVE system, the present study examines animal–computer interaction as a tool for advancing welfare through the lens of personality. Specifically, it investigates how trainer-assessed personality traits relate to gameplay performance in California sea lions during computerized enrichment. By connecting personality structure to device-logged measures of engagement, this study aims to demonstrate how animal–computer interaction can support individualized, animal-centered enrichment planning and contribute to environments where each animal can thrive [2,8,10,11].

The aim of this study was to investigate whether individual differences in personality are associated with variation in performance during computerized cognitive enrichment using the Enclosure Video Enrichment (EVE) system. It was anticipated that personality traits would correspond to distinct task performance patterns such as engagement, persistence, and efficiency. Based on previous findings linking temperament to cognitive style and problem-solving success across animal species, we hypothesized that personality traits in California sea lions would be associated with characteristic performance styles during enrichment tasks [23,29,32,33].

## 2. Materials and Methods

### 2.1. Animal Participants and Housing

This study was conducted with ten adult male California sea lions (*Zalophus californianus*) housed at the U.S. Navy Marine Mammal Program (MMP) in San Diego, California. At the time of data collection, animals ranged in age from 10 to 21 years (Table 1). All individuals had previously completed the six training phases of the Enclosure Video Enrichment (EVE) system, ensuring comparable familiarity with the device [14].

Sea lions were housed in floating, netted sea pens measuring 9 m^2^, each with a dry haul-out platform. Animals were rotated across conspecifics on a routine basis, providing opportunities for varied social interaction. All housing met or exceeded U.S. federal animal welfare requirements, and the MMP is accredited by the Association for Assessment and Accreditation of Laboratory Animal Care (AAALAC).

Participation in this project did not require procedures outside of standard training and enrichment activities. During EVE sessions, animals voluntarily hauled out onto the dry platform portion of their enclosure to interact with the device. Research approval was obtained through the MMP Institutional Animal Care and Use Committee (IACUC Protocol #139-2020) and the Navy Bureau of Medicine and Surgery (Navy Research Database #1245).

### 2.2. Personality Assessments

To evaluate individual differences in personality, animals were scored using a modified version of surveys conducted by Ciardelli and colleagues [1] which had been previously validated with California sea lions. Surveys were distributed to 22 professional trainers employed at the MMP. Trainers were eligible to complete surveys only if they had at least six months of direct experience working with a given animal, ensuring that ratings were based on extended observation and familiarity rather than brief or incidental encounters. Not every trainer rated every animal; instead, trainers evaluated only those sea lions for which they met the experience requirement. Each animal was rated by between four and ten trainers, providing multiple informed perspectives per individual. Ratings from all trainers were combined to calculate a mean value for each trait per animal. These mean values were then used for cluster analysis. In total, 62 surveys were completed across the ten sea lions. The surveys included 61 personality descriptors developed for marine mammal research, covering a broad range of temperament, social behavior, and cognitive tendencies [1]. Trainers scored each trait on a 5-point Likert-type scale, where 1 indicated “not at all like this animal” and 5 indicated “very much like this animal.” The use of multiple raters per animal minimized potential bias from individual observer perspectives and provided a reliable assessment of stable characteristics.

### 2.3. Interrater Reliability

Consistency across raters was examined using intraclass correlation coefficients (ICCs). Both ICC(3,1), which provides an estimate of reliability for single ratings, and ICC(3,k), which provides an estimate of reliability for mean scores across multiple raters, were calculated [34]. This approach has been applied in animal personality research to assess the extent to which traits may be considered reliably rated when multiple observers provide judgments [35].

### 2.4. Gameplay Data Collection

The initial training game comprised six sequential phases that progressively increased in difficulty and precision requirement [14]. During the earliest phase, animals were reinforced for pressing buttons while visually tracking the on-screen cursor. As training progressed, targets were introduced, and the placement and directional requirements of those targets became increasingly complex. Across phases, both the targets and the cursor gradually decreased in size to promote finer motor control. By Phase 6, animals demonstrated precise, independent control of the cursor to acquire targets without trainer guidance. The EVE system automatically recorded detailed interaction data during Phase 6 gameplay sessions. These data were exported from Microsoft Excel and subsequently analyzed in IBM SPSS Statistics (Version 29.0.1). Eight output variables were selected for inclusion in the analysis:

- Latency to target acquisition (s): first session latency, last session latency, mean latency of all sessions, and change in latency (first–last).
- Button press activity: first session total presses, last session total presses, mean presses, and change in presses (first–last).

Although measures such as number of sessions to criterion or days to acquisition were available, they were excluded as they were substantially affected by trainer-controlled factors (e.g., session length, reinforcement density, husbandry demands). Emphasis was instead placed on system-recorded variables from the EVE system, which provided consistent, objective records of performance in line with recommendations for accuracy and replicability [36,37,38].

### 2.5. Hierarchical Clustering

Hierarchical clustering analysis was performed using Ward’s [39] method to identify natural groupings of personality traits based on the similarity of their scores across animals. Ward’s method was selected because it minimizes within-cluster variance and performs well with small to moderate sample sizes [39,40,41]. Although this method can be sensitive to outliers, all animals were retained to preserve meaningful individual differences. Its use is also well established in animal behavior research identifying behavioral or personality groupings, where hierarchical clustering with Ward’s linkage produced compact and interpretable solutions [42,43,44]. Cluster selection was based on visual inspection of the dendrogram using a Euclidian distance cut of 13 to balance interpretability with internal cohesion [41] and aligns with practices in animal personality research that emphasize deriving multidimensional yet manageable groupings for practical application [25]. Analysis was conducted in SPSS Version 29.0.1.0 and employed Euclidean distance as the measure of similarity.

### 2.6. Correlation Analysis

The analysis proceeded in three sequential phases to examine relationships at different levels of personality organization. First, cluster-to-gameplay relationships were analyzed to identify broad behavioral patterns. Then, individual trait-to-gameplay correlations were investigated to detect more specific associations.

Both Kendall’s tau-b and Spearman’s rho correlation coefficients were employed across both analytical phases. This dual-method approach was implemented for several reasons: (1) Kendall’s tau-b is robust for small samples and tied ranks [45,46,47], (2) Spearman’s rho provides complementary effect size estimates that are more widely reported in psychological research [47] and (3) comparing results across methods enhances interpretive confidence when findings converge [48]. Individual trait examination allowed for detection of specific associations that might be obscured by aggregate measures [49]. This multilevel approach aligns with contemporary personality research, which emphasizes examining both hierarchical and component-level relationships [50]. Given the exploratory nature of the comparisons, significant results were interpreted cautiously, with emphasis placed on effects consistent across both correlation methods.

## 3. Results

### 3.1. Personality Clusters

**Figure 3:**
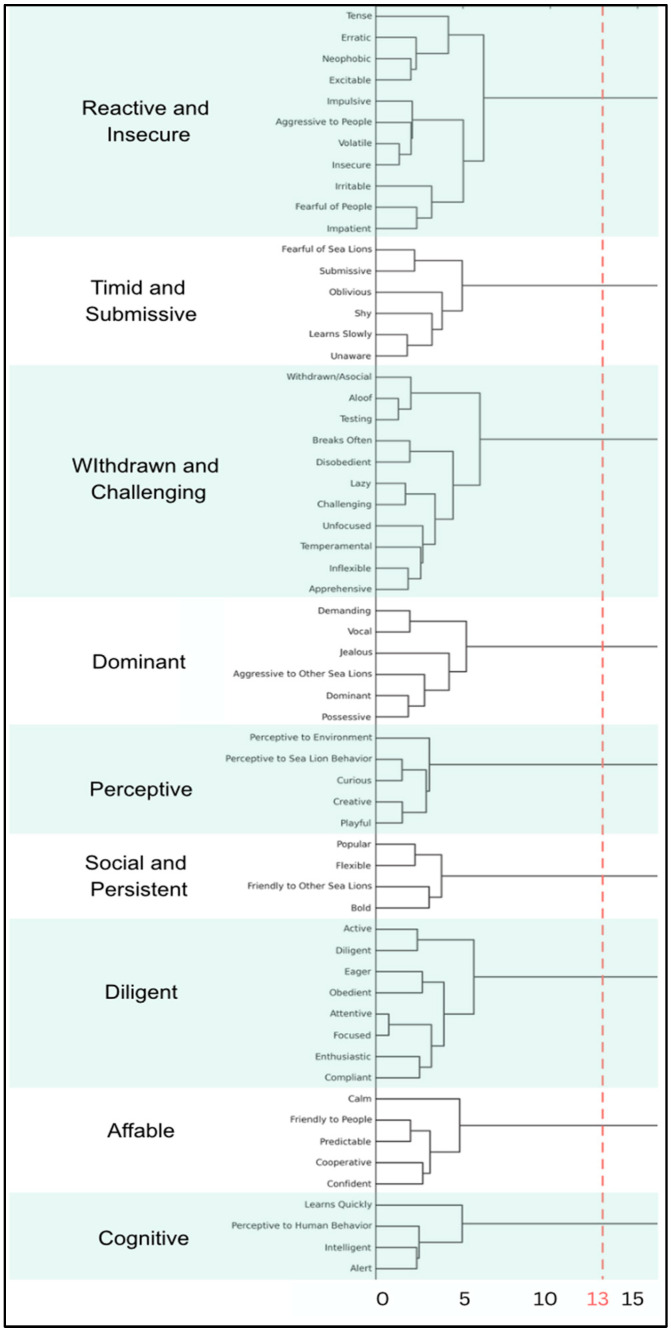
Partial dendrogram of all rated traits, showing cluster groupings based on a Euclidian distance cut of 13, shown with a dashed red line.

Ward’s [39] hierarchical clustering method was applied to the 61 rated personality traits using Euclidean distance as the measure of dissimilarity. Interpretation of the dendrogram yielded nine personality clusters (Figure 3). The clusters were named based on thematic commonalities among grouped traits, resulting in the following interpretable categories: Affable, Cognitive, Diligent, Perceptive, Reactive/Insecure, Social/Persistent, Timid/Submissive, Withdrawn/Challenging, and Dominant (Table 2).

### 3.2. Distinct Personality Profiles

**Figure 4:**
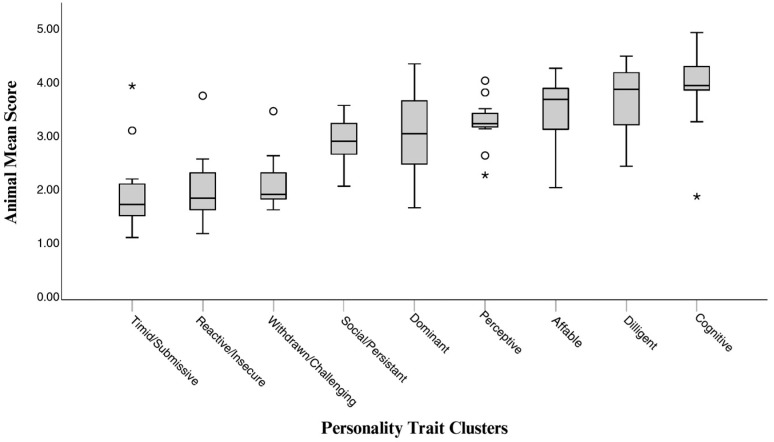
Boxplot of animal mean scores across nine personality trait clusters. Each data point represents an individual sea lion’s mean score for all traits assigned within that cluster. Note. ° = mild outlier (1.5–3 × IQR); * = extreme outlier (>3 × IQR).

The distribution of mean trait scores across the nine personality clusters is shown in Figure 4. Median scores were highest in the Cognitive, Diligent, and Affable clusters, intermediate in the Perceptive, Social & Persistent, and Dominant clusters, and lowest in the Timid/Submissive, Reactive & Insecure, and Withdrawn & Challenging clusters. Boxplot distributions also indicated variation in score ranges, with wider interquartile ranges in the Dominant, Affable, and Diligent clusters and narrower ranges in the Perceptive and Cognitive clusters. Several clusters contained outliers.

Exact mean values for each animal across the nine personality clusters are reported in Table 3. Overall, the profiles revealed a general tendency toward higher scores in the Cognitive, Diligent, and Affable clusters, and lower scores in Timid/Submissive, Reactive & Insecure, and Withdrawn & Challenging, though distinct individual patterns emerged. ANK scored high in Cognitive and Diligent traits, paired with low ratings in Timid/Submissive and Reactive & Insecure. ARU showed strong Cognitive, Diligent, and Affable scores. BER’s ratings were well-balanced, with elevated Diligent and Cognitive traits and relatively low Timid/Submissive tendencies. JCK demonstrated high Affable and Diligent traits with low Reactivity. JTY exhibited higher Timid/Submissive and lower Cognitive scores. MRT consistently scored high in Diligent and Cognitive traits, while REX showed strength in Affable and Cognitive but lower Diligent scores. SLD displayed moderate Affable and Cognitive ratings alongside higher Withdrawn & Challenging tendencies. TPO exhibited the most variability, with high Cognitive and Diligent traits but lower Affable and Timid/Submissive, alongside elevated Reactive & Insecure and Dominant scores. YOD showed high Diligent and Affable scores, coupled with relatively higher Timid/Submissive values.

**Figure 5:**
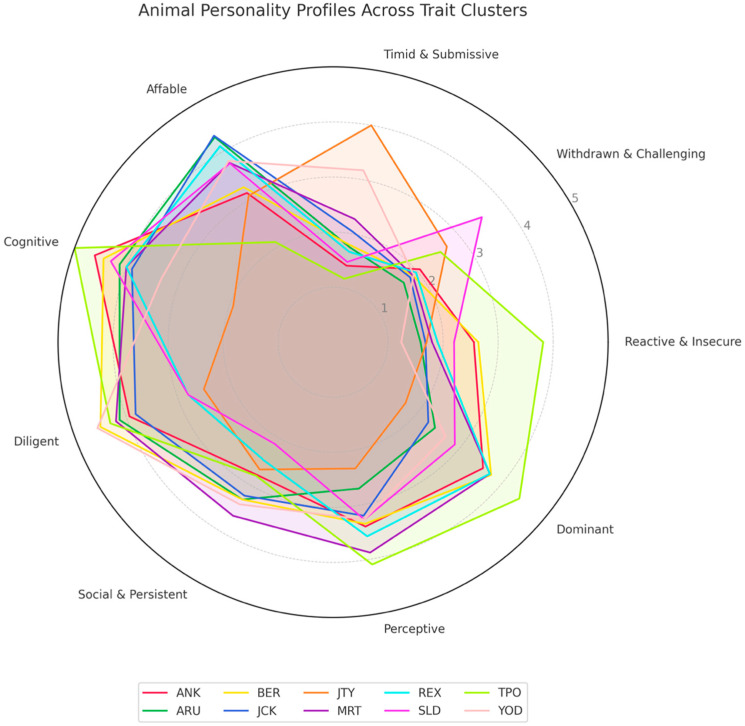
Radar chart of individual sea lion personality profiles across nine clusters, illustrating relative strengths in different trait groupings rather than exclusive cluster membership. Interior circles represent mean scores for each animal of all traits for assigned to each cluster.

While the boxplot (Figure 4) summarizes group-level trends, the radar chart (Figure 5) illustrates individual variation across clusters. It depicts distinct personality profiles for the nine sea lions, showing relative strengths and weaknesses across clusters rather than exclusive membership.

### 3.3. Correlations Between Personality Clusters and Gameplay Variables

Analysis of the correlations between personality clusters and gameplay variables revealed several significant associations (see Appendix A). Although Spearman’s rho consistently produced larger coefficients than Kendall’s tau-b, both methods identified the same set of significant associations, indicating that the results were robust to the choice of correlation method. Higher scores in the *Perceptive* (ρ = −0.733, *p* = 0.016; τ = −0.600, *p* = 0.016) and *Dominant* (ρ = −0.742, *p* = 0.014; τ = −0.584, *p* = 0.020) clusters were strongly linked to faster response times during the initial session, as reflected in significant negative correlations with first session latency. The *Perceptive* cluster also showed a significant negative association with last session latency (τ = −0.511, *p* = 0.040), indicating that more perceptive individuals sustained quicker response times throughout the training phase. The *Affable* cluster was positively associated with reduction in latency over time (ρ = 0.709, *p* = 0.022; τ = 0.556, *p* = 0.025), indicating that animals with higher affability scores showed greater improvements in response speed across sessions. Additionally, higher scores in the *Dominant* cluster were significantly related to fewer button presses during the first session (ρ = −0.723, *p* = 0.018; τ = −0.584, *p* = 0.020). Other correlations between personality clusters and gameplay variables were not statistically significant.

### 3.4. Correlations Between Individual Traits and Gameplay Variables

Following the analysis of trait clusters, individual traits were examined to identify more specific associations with gameplay variables that may have been obscured when traits were aggregated. Analyses using Kendall’s tau and Spearman’s rho identified several significant and highly significant associations between personality traits and gameplay measures (Latency, Table 4; Button Presses, Table 5).

For latency, *Calm* was significant in both Kendall’s tau and Spearman’s rho, showing positive correlations with first session latency (τ = 0.511, *p* = 0.040; ρ = 0.648, *p* = 0.043) and change in latency (τ = 0.556, *p* = 0.025; ρ = 0.648, *p* = 0.043). This indicates that calm animals were slower to begin responding and became even slower across sessions. *Challenging* was also significant in both tests, with negative correlations for mean latency (τ = −0.539, *p* = 0.031; ρ = −0.699, *p* = 0.024) and change in latency (τ = −0.539, *p* = 0.031; ρ = −0.657, *p* = 0.039), suggesting that more challenging animals were faster overall and improved across sessions. *Compliant* was significant only in Spearman’s rho, showing a positive correlation with mean latency (ρ = 0.632, *p* = 0.050) and a negative correlation with change in button presses (ρ = −0.632, *p* = 0.050), reflecting slower overall responses but improved efficiency over time.

Several traits were linked to faster responding. *Erratic* was significant in both Kendall’s tau and Spearman’s rho, with negative correlations for first session latency (τ = −0.523, *p* = 0.038; ρ = −0.640, *p* = 0.046) and change in latency (τ = −0.659, *p* = 0.009; ρ = −0.768, *p* = 0.009). Both tests reached high significance for change in latency (*p* < 0.01). *Perceptive to Sea Lion Behavior* showed the strongest effects, being highly significant in both tests for first session latency (τ = −0.659, *p* = 0.009; ρ = −0.823, *p* = 0.003) and change in latency (τ = −0.614, *p* = 0.015; ρ = −0.793, *p* = 0.006). *Playful* was significant in both tests for first session latency (τ = −0.535, *p* = 0.036; ρ = −0.665, *p* = 0.036), indicating quicker initial responses. *Predictable* was significant only in Spearman’s rho, with a positive correlation to first session latency (ρ = 0.636, *p* = 0.048), suggesting slower initial responding.

For button-press measures, *Dominant* was significant in both Kendall’s tau and Spearman’s rho, showing negative correlations with mean button presses (τ = −0.584, *p* = 0.020; ρ = −0.718, *p* = 0.020), indicating greater efficiency. In contrast, *Friendly to Other Sea Lions* and *Shy* were significant in both tests, with positive correlations to button presses (τ = 0.494, *p* = 0.048; ρ = 0.669, *p* = 0.035), suggesting reduced efficiency. *Aloof* was also significant in both tests, with strong positive correlations to first session button presses (τ = 0.685, *p* = 0.029; ρ = 0.685, *p* = 0.029), indicating higher initial but less efficient engagement. *Fearful of Sea Lions* (ρ = 0.669, *p* = 0.035) and *Obedient* (ρ = 0.646, *p* = 0.043) were significant only in Spearman’s rho, both showing reduced efficiency due to more button presses.

Several traits showed significance only in Spearman’s rho. Impulsive (ρ = −0.638, *p* = 0.047), Enthusiastic (ρ = −0.669, *p* = 0.034), and Possessive (ρ = −0.644, *p* = 0.044) were linked to fewer mean button presses, indicating greater efficiency. In contrast, Fearful of Sea Lions (ρ = 0.669, *p* = 0.035) and Obedient (ρ = 0.646, *p* = 0.043) were associated with more presses, reflecting lower efficiency. Compliant (ρ = −0.632, *p* = 0.050) was related to change in button presses, with higher scores corresponding to smaller reductions across sessions. None of these associations were significant in Kendall’s tau.

## 4. Discussion

The findings suggest that personality meaningfully shapes how animals interact with computerized tasks, reinforcing the value of individual differences in enrichment design. It contributes to the personality–cognition literature by aligning trait-based profiles with engagement patterns in a managed context, without overextending specific claims [23,28,51]. It also situates a novel cognitive enrichment modality for marine mammals within a welfare-oriented framework, consistent with recommendations for individualized, adaptive design [2,8].

### 4.1. Trainer Ratings Are Reliable, Useful, and Actionable

Interrater reliability analyses indicated that trainer ratings were both reliable and consistent, supporting the use of structured caregiver judgments as valid, animal-centered inputs for welfare decision-making, including enrichment design [34,35,52]. Because raters knew the animals well, their assessments integrated day-to-day observations across contexts and complemented automated gameplay metrics. Anecdotally, trainers reported a clear sense of how their animals tend to play and engage with the device; aggregation of personality trait scores corroborated many of these expectations, lending empirical support to trainer insight [52]. This blend of gameplay metrics and trainer observations provide a practical basis for tailoring enrichment and spotting early signs of concern, so staff can adjust reinforcement, session structure, or environmental complexity before issues become entrenched [53,54].

An important consideration in interpreting these findings is the potential for inherent bias in trainer ratings. Because trainers develop strong relationships with the animals in their care, they may be inclined, consciously or unconsciously, to score individuals more favorably across traits. This tendency has been observed in other welfare rating contexts, where familiarity and emotional investment can influence assessments [52,55]. In the present study, overall ratings trended toward positive evaluations, suggesting that while trainers provided consistent and actionable profiles, their judgments may overemphasize desirable qualities or underreport problematic tendencies. At the same time, a generally positive perspective can itself enhance welfare by supporting trust-based relationships, encouraging more patient training approaches, and reinforcing constructive human–animal interactions [56]. Thus, trainer bias should be acknowledged as a methodological limitation, but its welfare benefits as part of the human–animal bond should not be overlooked.

### 4.2. Clusters Reflect Complex, Multidimensional Profiles

Cluster analysis provided a useful middle level summary of personality organization, yet no animal fit neatly within a single cluster. Each individual expressed a distinctive mix of cluster scores, consistent with personality as a multidimensional construct in which factors describe covarying tendencies rather than fixed categories [23,57]. In practice, clusters give a simple shorthand for broad dispositions, and each animal’s combined cluster and trait scores provide the detail needed for case-by-case decisions. Framing personality in this way avoids a one size fits all approach and instead informs thoughtful planning that reflects the combination of strengths and challenges present in each individual.

### 4.3. Personality-Performance Associations

The analysis revealed clear links between personality and gameplay outcomes. Animals scoring higher in the Perceptive and Dominant clusters responded more quickly in their first session, and Perceptive animals maintained faster responding across later sessions. The Affable cluster was associated with improvement over time, with higher scores predicting greater reductions in latency. This pattern suggests that affable animals may begin cautiously but adjust well with repeated exposure. At the trait level, Calm animals were slower to start and became progressively slower, while Challenging animals grew faster and more efficient over time. Erratic and Playful traits were linked to quicker responding, whereas Friendly to Other Sea Lions and Shy were tied to higher button press totals, reflecting less efficient task engagement.

No single profile defined success; different dispositions supported different strategies. Affable animals demonstrated adaptability, Dominant animals showed early efficiency, and Perceptive animals sustained attentiveness. In contrast, traits tied to social sensitivity or hesitation reduced efficiency. These findings support previous research showing that stable individual differences predict problem solving across species [23,28,58,59]. Personality ratings therefore provide insight into likely response patterns and form a basis for tailoring enrichment approaches.

### 4.4. Personalizing Enrichment from Personality Profiles

Translating these results into practice, personality-linked profiles can guide enrichment choices so that activities align with each animal’s natural tendencies and preferred ways of interacting. Animals requiring more responses may benefit from tasks that reward rapid successive actions with short response windows [54,60]. Slower responders may need predictable cue timing, longer decision windows, and denser reinforcement early in sessions. Highly attentive animals can be challenged with discrimination-based tasks, while easily distracted or socially oriented individuals may engage better with simpler, predictable tasks introduced gradually [10,53]. For animals showing impulsivity, shorter bouts with clear trial boundaries and immediate reinforcement can maintain accuracy and motivation.

These adjustments keep enrichment achievable, motivating, and controllable, features linked to positive welfare outcomes [10,53]. They also reinforce the trainer–animal relationship, creating a positive feedback loop in which effective enrichment improves engagement, reduces stress, and strengthens bonds [52,56]. Overall, this study suggests that adaptive enrichment, in which task parameters, feedback, and progression respond to personality profiles, has strong potential to improve both engagement and welfare outcomes.

In the context of computerized cognitive enrichment, these findings indicate that the EVE system can operate as a flexible platform for individualized cognitive challenge. By integrating personality-informed data with gameplay metrics, parameters such as response timing, reinforcement density, and task complexity can be adjusted to match each animal’s behavioral profile. For example, more exploratory or persistent sea lions could be presented with progressively complex discriminations or variable ratio reinforcement to sustain engagement, whereas more cautious individuals may benefit from gradual task progression and predictable feedback that builds on confidence and motivation [10,54,60,61].

Adaptive calibration aligns with welfare-centered design principles that emphasize appropriate challenges and individual agency, to help ensure that enrichment remains achievable and rewarding across diverse personality types [62,63]. In practice, combining personality assessment with a responsive technological platform supports longer term engagement, sustained cognitive challenge, and effective trainer-animal interaction, while leveraging caregiver insight as valid input to enrichment planning [52,54].

### 4.5. Limitations and Future Directions

This study focused on Navy sea lions because they were already engaging with the EVE system as part of their regular enrichment and training routines. At the time, the EVE system was not yet available to other facilities, and including animals from zoos or aquariums would have introduced variability in housing, enrichment history, and human interaction. Future research should examine EVE performance across institutions once similar systems become more widely accessible to assess generalizability across managed-care contexts.

The small sample size and limited number of qualified trainers restricted the power of predictive analyses, so personality measures were primarily examined through correlational patterns. Expanding future work to include larger, more diverse populations could support predictive modeling and enhance the practical application of personality assessment for individualized enrichment. Finally, the absence of female sea lions, due to the Navy’s all-male operational cohort, limits generalization across sexes; future studies including females could clarify whether the observed personal-performance associations hold more broadly.

While recent research shows that technologies like the EVE system can enhance welfare, participation, and health outcomes [16,17], these systems require additional resources, technical expertise, and ongoing maintenance. Not all facilities have access to the necessary infrastructure, and ensuring the durability of technology in aquatic environments remains a practical concern. Importantly, trainers and animal care staff are often time constrained and over tasked, with surveys and qualitative studies consistently identifying lack of staff time and heavy workloads [64] as major barriers to the consistent implementation and evaluation of enrichment, including cognitive enrichment, in zoos and aquariums. The additional time required to research, plan, deliver, observe, and adjust enrichment, especially when using new technology, can make it difficult for staff to integrate these systems into already demanding daily routines. As a result, even when technological enrichment is available, staff capacity and competing priorities can limit its consistent use and the ability to fully realize its benefits for animal welfare.

Gameplay and performance data were limited by pre-designed outputs already imbedded into the EVE design [14]. It would be of interest to investigate additional gameplay variables, physiological outputs such as heart rate, or whether other factors influence performance, such as housed conspecifics or trainers present.

Finally, beyond animal welfare, technology-based enrichment may offer additional benefits to zoos and aquariums by showcasing animals’ cognitive abilities and leveraging a “coolness factor” that can increase public interest and engagement [65].

## 5. Conclusions

This study demonstrates that personality traits significantly influence how California sea lions engage with computerized cognitive enrichment. Trainer ratings provided reliable insights, and gameplay measures captured meaningful individual differences. Together, these findings support the value of tailoring enrichment to personality, moving beyond generic designs toward adaptive strategies that optimize welfare and engagement. While small sample size and technological constraints limit generalizability, the results highlight both the promise of adaptive enrichment systems and the practical challenges of their implementation. Ultimately, integrating personality assessment with cognitive technology offers a powerful approach to individualized enrichment in managed care, with benefits for both animal welfare and institutional missions.

## Figures and Tables

**Table 1:** Age of the ten California sea lions and the number of trainers who completed personality surveys for each individual.

Animal ID	Age	Number of Trainers Surveyed
ANK	14	9
ARU	10	4
BER	13	5
JCK	19	7
JTY	14	4
MRT	11	6
REX	19	9
SLD	20	10
TPO	13	4
YOD	14	4

**Table 2:** Personality trait clusters derived from Ward’s hierarchical clustering, showing the grouping of individual traits into nine broader categories.

Personality Trait Clusters		
**Reactive and Insecure**	Aggressive to People Volatile Tense Irritable Erratic Fearful of People	Impulsive Neophobic Impatient Excitable Insecure
**Withdrawn and Challenging**	Aloof Withdrawn/Asocial Inflexible Temperamental Apprehensive Lazy	Unfocused Breaks Often Challenging Disobedient Testing
**Timid and Submissive**	Learns Slowly Unaware Oblivious	Fearful of Sea Lions Shy Submissive
**Affable**	Cooperative Friendly to People Calm	Predictable Confident
**Cognitive**	Intelligent Learns Quickly Alert	Perceptive to Human Behavior
**Diligent**	Eager Enthusiastic Complaint Obedient	Attentive Active Diligent Focused
**Social and Persistent**	Friendly to Other Sea Lions Popular	Bold Flexible
**Perceptive**	Perceptive to Sea Lion Behavior Perceptive to Environment	Creative Curious Playful
**Dominant**	Aggressive to Other Sea Lions Dominant Jealous	Possessive Demanding Vocal

**Table 3:** Mean Personality trait cluster scores for nine sea lions.

Animal ID	Reactive/ Insecure	Withdrawn/ Challenging	Timid/ Submissive	Affable	Cognitive	Dilligent	Social/Persistant	Perceptive	Dominant
ANK	2.56	2.06	1.41	3.13	4.61	3.94	2.78	3.40	3.56
ARU	1.59	1.68	1.75	4.30	4.13	4.13	3.30	2.70	2.42
BER	2.64	1.89	1.79	3.25	4.44	4.50	3.30	3.35	3.75
JCK	1.68	1.83	2.04	4.33	3.89	3.82	3.22	3.20	2.26
JTY	1.70	2.70	4.00	3.07	1.93	2.50	2.67	2.33	1.72
MRT	1.80	1.89	2.27	3.76	4.00	4.20	3.64	3.88	3.72
REX	1.90	1.97	1.67	4.11	4.00	2.80	2.49	3.58	3.72
SLD	2.20	3.53	1.48	3.75	4.30	2.80	2.13	3.27	2.89
TPO	3.82	2.55	1.17	2.10	5.00	4.31	2.80	4.10	4.42
YOD	1.24	1.91	3.17	3.80	3.33	4.56	3.40	3.27	2.67

**Table 4:** Significant relationships between individual traits and latency variables. Correlations are reported with Spearman’s rho (ρ) and Kendall’s tau (τ), with associated *p* values.

Trait	First Session Latency (ρ)	First Session Latency (τ)	Last Session Latency (ρ)	Last Session Latency (τ)	Mean Latency (ρ)	Mean Latency (τ)	Change in Latency (ρ)	Change in Latency (τ)
Calm	ρ = 0.648 (*p* = 0.043)	τ = 0.511 (*p* = 0.040)			ρ = −0.699 (*p* = 0.024)	τ = −0.539 (*p* = 0.034)	ρ = 0.648 (*p* = 0.043)	τ = 0.556 (*p* = 0.025)
Challenging					ρ = 0.632 (*p* = 0.050)		ρ = −0.657 (*p* = 0.039)	τ = −0.539 (*p* = 0.031)
Compliant								
Erratic	ρ = −0.640 (*p* = 0.045)	τ = −0.523 (*p* = 0.038)					ρ = −0.768 (*p* = 0.009)	τ = −0.659 (*p* = 0.009)
Perceptive to Sea Lion Behavior	ρ = −0.823 (*p* = 0.006)	τ = −0.659 (*p* = 0.009)					ρ = −0.793 (*p* = 0.006)	τ = −0.614 (*p* = 0.015)
Playful	ρ = −0.665 (*p* = 0.038)	τ = −0.535 (*p* = 0.036)						
Predictable	ρ = 0.636 (*p* = 0.048)							

**Table 5:** Significant relationships between individual traits and button-press variables. Correlations are reported with Spearman’s rho (ρ) and Kendall’s tau (τ), with associated *p* values.

Trait	First Session Mean Button Presses (ρ)	First Session Mean Button Presses (τ)	Last Session Mean Button Presses (ρ)	Last Session Mean Button Presses (τ)	Mean Button Presses (ρ)	Mean Button Presses (τ)	Change in Button Presses (ρ)	Change in Button Presses (τ)
Compliant					ρ = −0.718 (*p* = 0.020)	τ = −0.584 (*p* = 0.020)	ρ = −0.632 (*p* = 0.050)	
Dominant					ρ = 0.669 (*p* = 0.035)	τ = 0.494 (*p* = 0.048)		
Shy			ρ = 0.669 (*p* = 0.035)	τ = 0.494 (*p* = 0.048)				
Aloof	ρ = 0.685 (*p* = 0.029)	τ = 0.685 (*p* = 0.029)						
Impulsive	ρ = −0.638 (*p* = 0.047)							
Enthusiastic							ρ = −0.669 (*p* = 0.034)	
Fearful of Sea Lions					ρ = 0.669 (*p* = 0.035)			
Obedient					ρ = 0.646 (*p* = 0.043)			
Possessive							ρ = −0.644 (*p* = 0.044)	

## Data Availability

Full data available upon request.

## Appendix A

**Table A1:** Spearman’s rho correlations between personality clusters and gameplay variables. Values represent correlation coefficients.

Correlations											
			Reactive and Insecure	Withdrawn and Challenging	Timid and Submissive	Affable	Cognitive	Dilligent	Social and Persistant	Perceptive	Dominant
Spearman’s rho	Mean Latency	Correlation Coefficient	−0.491	−0.0394	−0.067	0.406	−0.097	0.164	0.134	−0.370	−0.377
		Sig. (2−tailed)	0.150	0.260	0.855	0.244	0.789	0.650	0.713	0.293	0.283
		N	10	10	10	10	10	10	10	10	10
	First Session Latency	Correlation Coefficient	−0.515	−0.285	0.091	0.539	−0.219	−0.280	−0.109	−0.733 *	−0.742 *
		Sig. (2−tailed)	0.128	0.425	0.803	0.108	0.544	0.434	0.763	0.016	0.014
		N	10	10	10	10	10	10	10	10	10
	Last Session Latency	Correlation Coefficient	−0.382	−0.018	0.067	−0.006	−0.097	0.201	0.079	−0.539	−0.401
		Sig. (2−tailed)	0.276	0.960	0.855	0.987	0.789	0.578	0.828	0.108	0.250
		N	10	10	10	10	10	10	10	10	10
	Change in latency	Correlation Coefficient	−0.442	−0.455	−0.091	0.709 *	−0.067	−0.140	−0.012	−0.442	−0.492
		Sig. (2−tailed)	0.200	0.187	0.803	0.022	0.854	0.700	0.973	0.200	0.148
		N	10	10	10	10	10	10	10	10	10
	Mean Number of Button Presses per level	Correlation Coefficient	−0.552	−0.297	0.479	0.176	−0.407	0.304	0.553	−0.297	−0.468
		Sig. (2−tailed)	0.098	0.405	0.162	0.627	0.243	0.393	0.097	0.405	0.172
		N	10	10	10	10	10	10	10	10	10
	First Mean Button Presses	Correlation Coefficient	−0.467	0.164	0.358	0.042	−0.468	−0.201	−0.073	−0.624	−0.723 *
		Sig. (2−tailed)	0.174	0.651	0.310	0.907	0.172	0.578	0.841	0.054	0.018
		N	10	10	10	10	10	10	10	10	10
	Last Mean Button Presses	Correlation Coefficient	−0.370	−0.042	0.321	−0.139	−0.310	0.255	0.176	−0.491	−0.383
		Sig. (2−tailed)	0.293	0.907	0.365	0.701	0.383	0.476	0.626	0.150	0.275
		N	10	10	10	10	10	10	10	10	10
	Change in Mean Button Presses	Correlation Coefficient	−0.273	0.248	0.358	−0.030	−0.407	−0.413	−0.109	−0.394	−0.614
		Sig. (2−tailed)	0.446	0.489	0.310	0.934	0.243	0.235	0.763	0.260	0.059
		N	10	10	10	10	10	10	10	10	10

*. Correlation is significant at the 0.05 level (2-tailed).

**Table A2:** Kendall’s tau-b correlations between personality clusters and gameplay variables. Values represent correlation coefficients.

Correlations											
			Reactive and Insecure	Withdrawn and Challenging	Timid and Submissive	Affable	Cognitive	Dilligent	Social and Persistant	Perceptive	Dominant
Kendall’s tau_b	Mean Latency	Correlation Coefficient	−0.333	−0.378	−0.022	0.200	−0.045	0.180	0.135	−0.289	−0.270
		Sig. (2−tailed)	0.180	0.128	0.929	0.421	0.857	0.472	0.590	0.245	0.281
		N	10	10	10	10	10	10	10	10	10
	First Session Latency	Correlation Coefficient	−0.378	−0.156	0.111	0.422	−0.180	−0.180	−0.090	−0.600 *	−0.584 *
		Sig. (2−tailed)	0.128	0.531	0.655	0.089	0.472	0.472	0.719	0.016	0.020
		N	10	10	10	10	10	10	10	10	10
	Last Session Latency	Correlation Coefficient	−0.200	−0.067	0.022	−0.022	−0.090	0.090	0.090	−0.511 *	−0.315
		Sig. (2−tailed)	0.421	0.788	0.929	0.929	0.719	0.719	0.719	0.040	0.209
		N	10	10	10	10	10	10	10	10	10
	Change in latency	Correlation Coefficient	−0.244	−0.289	−0.111	0.556 *	−0.045	−0.045	−0.045	−0.378	−0.449
		Sig. (2−tailed)	0.325	0.245	0.655	0.025	0.857	0.857	0.857	0.128	0.072
		N	10	10	10	10	10	10	10	10	10
	Mean Number of Button Presses per level	Correlation Coefficient	−0.333	−0.200	0.333	0.111	−0.270	0.135	0.405	−0.200	−0.315
		Sig. (2−tailed)	0.180	0.421	0.180	0.655	0.281	0.590	0.106	0.421	0.209
		N	10	10	10	10	10	10	10	10	10
	First Mean Button Presses	Correlation Coefficient	−0.333	0.156	0.244	0.022	−0.270	−0.135	−0.045	−0.467	−0.584 *
		Sig. (2−tailed)	0.180	0.531	0.325	0.929	0.281	0.590	0.857	0.060	0.020
		N	10	10	10	10	10	10	10	10	10
	Last Mean Button Presses	Correlation Coefficient	−0.200	−0.067	0.200	−0.111	−0.180	0.225	0.180	−0.422	−0.225
		Sig. (2−tailed)	0.421	0.788	0.421	0.655	0.472	0.369	0.472	0.089	0.369
		N	10	10	10	10	10	10	10	10	10
	Change in Mean Button Presses	Correlation Coefficient	−0.244	0.156	0.244	−0.067	−0.270	−0.315	−0.045	−0.289	−0.405
		Sig. (2−tailed)	0.325	0.531	0.325	0.788	0.281	0.209	0.857	0.245	0.106
		N	10	10	10	10	10	10	10	10	10

*. Correlation is significant at the 0.05 level (2-tailed).
